# MYTHEN III: advancements in single photon counting detectors for synchrotron powder diffraction experiments

**DOI:** 10.1107/S1600577525000438

**Published:** 2025-02-13

**Authors:** Marie Andrä, Anna Bergamaschi, Filippo Baruffaldi, Martin Brückner, Maria Carulla, Nicola Casati, Antonio Cervellino, Roberto Dinapoli, Erik Fröjdh, Dominic Greiffenberg, Shqipe Hasanaj, Julian Heymes, Viktoria Hinger, Pawel Kozlowski, Carlos Lopez Cuenca, Dominik Meister, Davide Mezza, Konstantinos Moustakas, Aldo Mozzanica, Kirsty Paton, Christian Ruder, Valerio Scagnoli, Grigory Smolentsev, Bernd Schmitt, Dhanya Thattil, Xiangyu Xie, Jiaguo Zhang

**Affiliations:** a Paul Scherrer Insitute, Forschungsstrasse 111, 5232Villigen – PSI, Switzerland; b Laboratory for Mesoscopic Systems, Department of Materials ETH Zürich Zürich Switzerland; University of Malaga, Spain

**Keywords:** single photon counting, microstrip sensors, X-ray detectors, X-ray powder diffraction, MYTHEN III

## Abstract

The single photon counting microstrip detector MYTHEN III was developed at the Paul Scherrer Institute to satisfy the increasing demands in detector performance of synchrotron radiation experiments, focusing on time-resolved and on-edge powder diffraction measurements. Similar to MYTHEN II, the detector installed on the Material Science beamline covers 120° in 2θ. It is based on the MYTHEN III.0 readout chip wire-bonded to silicon strip sensors with a pitch of 50 µm, and it provides improved performance and features with respect to the previous version. Taking advantage of the three independent comparators of MYTHEN III, it is possible to obtain an improvement in the maximum count rate capability of the detector at 90% efficiency from 2.9 ± 0.8 Mphotons s^−1^ strip^−1^ to 11 ± 2 Mphotons s^−1^ strip^−1^ thanks to the detection of pile-up at high photon flux. The readout chip offers additional operation modes such as pump–probe and digital on-chip interpolation. The maximum frame rate is up to 360 kHz in 8-bit mode with dead-time-free readout. The minimum detectable energy of MYTHEN III is 4.3 ± 0.3 keV with a minimum equivalent noise charge (ENC) of 121 ± 8 electrons and a threshold dispersion below 33 ± 10 eV. The energy calibration is affected by temperature by less than 0.5% °C^−1^. This paper presents a comprehensive overview of the MYTHEN III detector system with performance benchmarks, and highlights the improvements reached in powder diffraction experiments compared with the previous detector generation.

## Abstract 2

This paper describes in detail the upgraded MYTHEN III single photon counting microstrip detector developed for powder diffraction, and its performance.

## 1. Introduction

X-ray powder diffraction at synchrotrons is a critical technique for analyzing the composition of materials, determining crystal structures and observing how specimens respond to external stimuli. It allows for the nondestructive study of multicomponent materials and microcrystalline compounds. Experiments often rely on the comparison of strong and weak diffraction peaks at various scattering angles, hence requiring detectors with a high dynamic range, large angular coverage and high spatial resolution. In addition, detectors must offer fast frame rates and low noise levels, especially for pump–probe or *in situ* measurements. Since ideal powder samples contain all possible crystal orientations, only one-dimensional data need to be recorded to reconstruct the sample characteristics, reducing data throughput and allowing for time-resolved measurements if combined with fast frame rates of the detector (Willmott, 2019 ▸).

Hybrid pixel photon counting detectors like, for example, EIGER (Tinti *et al.*, 2015 ▸), EIGER 2 (Bochenek *et al.*, 2018 ▸; Donath *et al.*, 2023 ▸), the UFXC camera (Koziol *et al.*, 2018 ▸; Dawiec *et al.*, 2019 ▸) and MEDIPIX 3 (Ballabriga *et al.*, 2013 ▸) are also suitable for powder diffraction experiments. However, the MYTHEN III one-dimensional strip detector, with fewer readout channels and finer segmentation, delivers faster frame rates, higher angular resolution and broader angular coverage at a lower cost, making it better suited to meeting experimental requirements (Fisch *et al.*, 2015 ▸). To cover the same area with more than 30000 channels in one direction and the same angular range, a pixel detector of almost five million pixels would be required. The material costs alone would amount to a few million CHF, not including the significantly higher expenses associated with the more powerful data backend required to support the higher data throughput.

This work describes the performance of the MYTHEN III detector, with a focus on the system installed on the Material Science (MS) beamline of the Swiss Light Source (SLS). Section 2 provides a detailed description of the detector. Section 3 presents the characterization measurements, defining the key performance metrics. In Section 4 the performance of MYTHEN III in powder X-ray diffraction experiments is compared with that of its predecessor, MYTHEN II. Section 5 discusses the performance of MYTHEN III as a beam and polarization monitor. The results are summarized in Section 6.

## 2. Detector description

The Microstrip sYstem for Time rEsolved experimeNts (MYTHEN) is developed for powder diffraction measurements at the Paul Scherrer Institute (PSI). The first version was installed at the Swiss Light Source (SLS) in 2001 (Schmitt *et al.*, 2004 ▸) and upgraded to MYTHEN II in 2007 (Bergamaschi *et al.*, 2010 ▸). The system features a single photon counting application-specific integrated circuit (ASIC) and silicon strip sensors with a 50 µm pitch.

Increasing demands for higher data quality – such as improved minimum detectable energy, reduced noise, enhanced count rate capability and faster frame rates – along with the aging of MYTHEN II led to the development of MYTHEN III. The two prototype readout chips are presented by Andrä *et al.* (2019*b* ▸) and Andrä *et al.* (2019*a* ▸), respectively, while the performance of the final detector is discussed in this paper.

MYTHEN III offers several improvements over MYTHEN II, including reduced noise and threshold dispersion, which enable the detection of lower energies and provide more consistent performance across all energy levels, as well as increased count rate capability, faster frame rate and higher dynamic range compared to MYTHEN II. One of its key advantages is the inclusion of three comparators and counters per channel, each with independent thresholds and gates. This design supports energy windowing, stroboscopic pump–probe experiments with up to three time slots, and enhanced count rate capability by tracking signal pile-up at high photon fluxes (Andrä *et al.*, 2019*a* ▸). Section 2.1 describes the ASIC, followed by the design of a module consisting of ten ASICs in Section 2.2. The detector system developed for the MS beamline at the SLS is covered in Section 2.3. Its design has been optimized to cover a solid angle of 120° without gaps in the diffraction pattern, and the process of mounting and replacing modules has been greatly simplified. The fully parallel readout of all modules allows for frame rates of up to 360 kHz.

### 2.1. The MYTHEN III.0 ASIC

**Figure 1:**
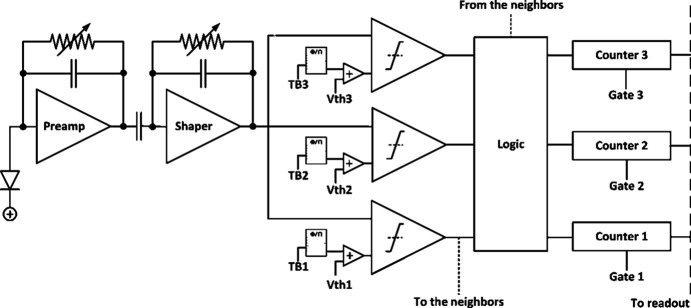
Scheme of a channel of the MYTHEN III chip.

The MYTHEN III.0 ASIC, developed at PSI, is manufactured in 110 nm UMC technology and features 128 channels. The architecture of a channel is illustrated in Fig. 1 ▸ (Andrä *et al.*, 2019*a* ▸). Each channel includes a charge-sensitive preamplifier and a shaper processing the signal from the sensor. These components come with adjustable feedback, allowing for tuning of the pulse shape, which in turn affects amplification and shaping time. Additionally, various capacitors in the feedback chain can be switched for the entire chip to modify the gain in discrete steps for both the preamplifier and the shaper. Lowering the gain results in increased speed and noise, creating a trade-off between the two. To accommodate different performance requirements, we have defined three main operating settings, *Standard*, *High Gain* and *Fast*, each with different noise and count rate capabilities.

The shaper output is fed into the three comparators per channel, each with its own individual threshold, defined by a reference voltage [*V*th_*N*_ (where *N* is the index of the comparator)] that is set globally for the entire module. Additionally, six trimbits (TB_*N*_) are available to fine-tune the comparator threshold, ensuring that manufacturing variations in the transistors are balanced and that the detector delivers a uniform response. When a photon signal exceeds the threshold in one of the comparators, the counting logic processes the event and distributes the count to one of the three gateable 24-bit counters.

The chip can operate in several modes, including standard, pump–probe, interpolation, and both analog and digital pulsing for testing. These modes can be selected by configuring the chip status register, which is also used to define the configuration of the feedback capacitors and the polarity of the comparator output.

In standard counting mode, the output of each comparator increments the corresponding counter during a defined exposure time. Each counter can be gated independently, either using three external signals or by defining the gate’s duration and delay in software. The thresholds of the comparators can also be adjusted individually for energy windowing or pile-up tracking (in which case the counters are typically enabled simultaneously).

In pump–probe mode, the output from a single comparator increments all three counters, depending on their individual gating. This mode is commonly used in stroboscopic experiments, where small mismatches between different time slots can hide minor variations in the comparators’ responses.

The interpolation mode is used to improve the detector’s spatial resolution and is fully detailed by Bergamaschi *et al.* (2022 ▸). It works by comparing the amplitude of the signal in a strip that detects a photon with the amplitudes in its neighboring strips. The count is then assigned to the left, right or central counter, depending on whether the signal was detected by the left neighbor, the right neighbor or neither. By using silicon sensors, it is possible to achieve an improvement in spatial resolution from 50 µm to 33 µm.

Additionally, both analog and digital pulsing modes can be used to pulse the preamplifier and counter, respectively, for testing purposes.

The number of counters per strip and the bit depth (8-, 16- or 24-bit) can be selected through different readout sequences. The readout of the chip is subdivided into groups of 32 strips which are read out serially.

### 2.2. The MYTHEN III module

**Figure 2:**
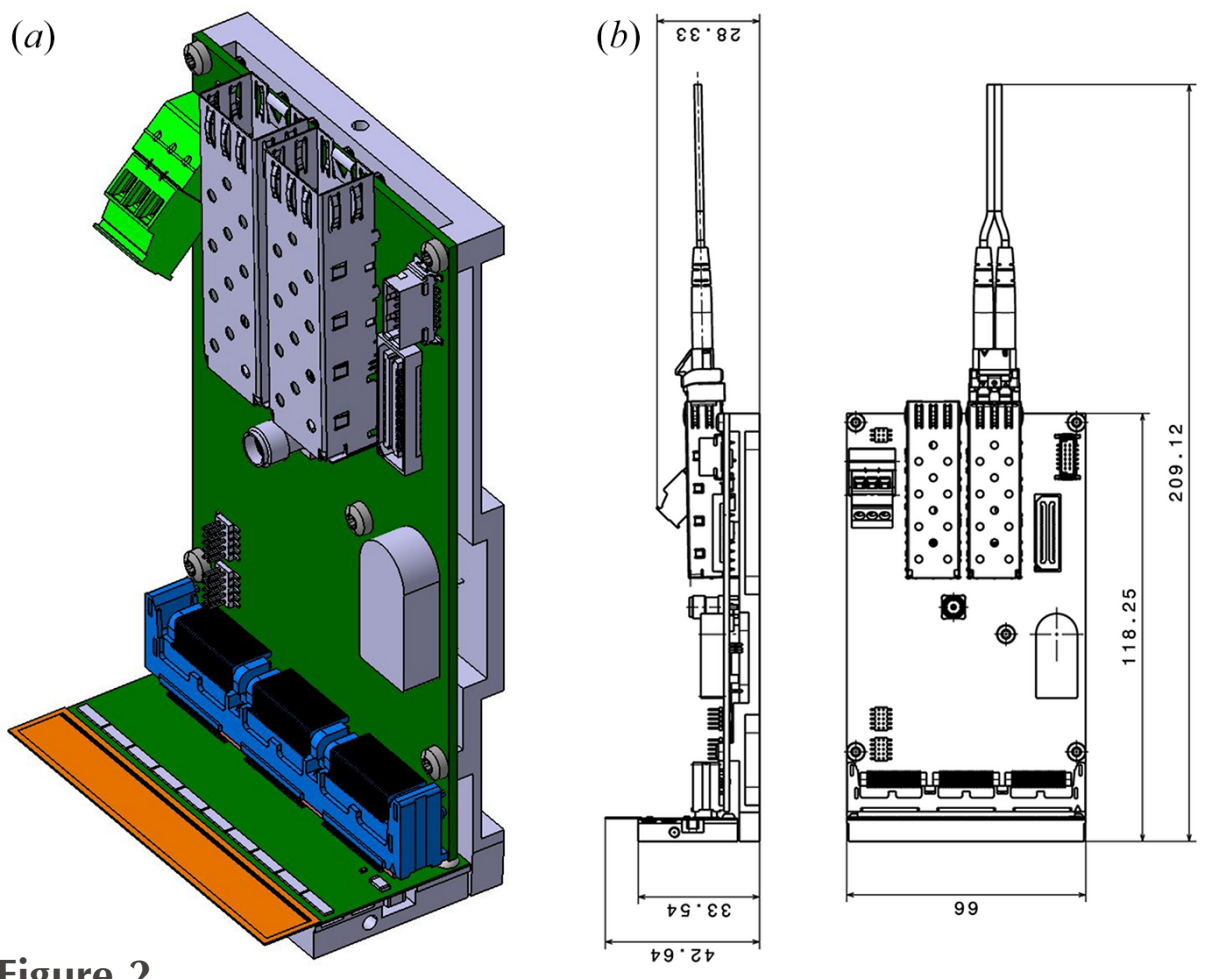
(*a*) Sketch of a MYTHEN III module with ten chips (gray) in rectangular alignment, a sensor (orange) and the readout board (green), and (*b*) the module dimensions.

Fig. 2 ▸ shows the design and dimensions of a MYTHEN III module. Each module contains ten ASICs glued to a custom high-density interconnect (HDI) and wire-bonded to a 64 mm-long silicon sensor, segmented into 1280 8 mm-long strips with a pitch of 50 µm. The sensor’s thickness can range from 300 µm to 650 µm, depending on the required quantum efficiency. The silicon sensors can also be replaced with high-*Z* materials, such as GaAs, for operation at high X-ray energies (Ruat *et al.*, 2018 ▸). Recently, full-size strip sensors (up to 640 mm × 4 mm) have been acquired and tests are foreseen in 2025, when SLS 2.0 comes into operation.

The module is connected to the readout board via an L-shaped connector, which improves assembly yield by separating the yield of the module from that of the readout board. This design also simplifies repairs and offers greater flexibility for future assemblies, where the connector could be flat or extended by a cable, *e.g.* when used in a vacuum environment.

All the chips in the module are controlled and read out in parallel through the readout board. For control purposes, a wired or fiber 1 Gbps Ethernet connection is used, while data are streamed using UDP over either the 1 Gbps control connection or a separate 10 Gbps fiber connection. The FPGA on the readout board is programmed to manage the detector and runs an embedded Linux system. In-house developed server software runs on the board, allowing the system to connect to the control PC via TCP/IP.

Thanks to its parallel design, several modules can be combined to create a larger detector system. One module is designated as the master, triggering the acquisition of the entire detector system via a flat band cable for synchronization. The master can receive four external TTL signals (one global trigger and one gate per counter) and outputs a busy signal during acquisition, along with three exposure signals (one for each counter).

### 2.3. MYTHEN III for X-ray powder diffraction

**Figure 3:**
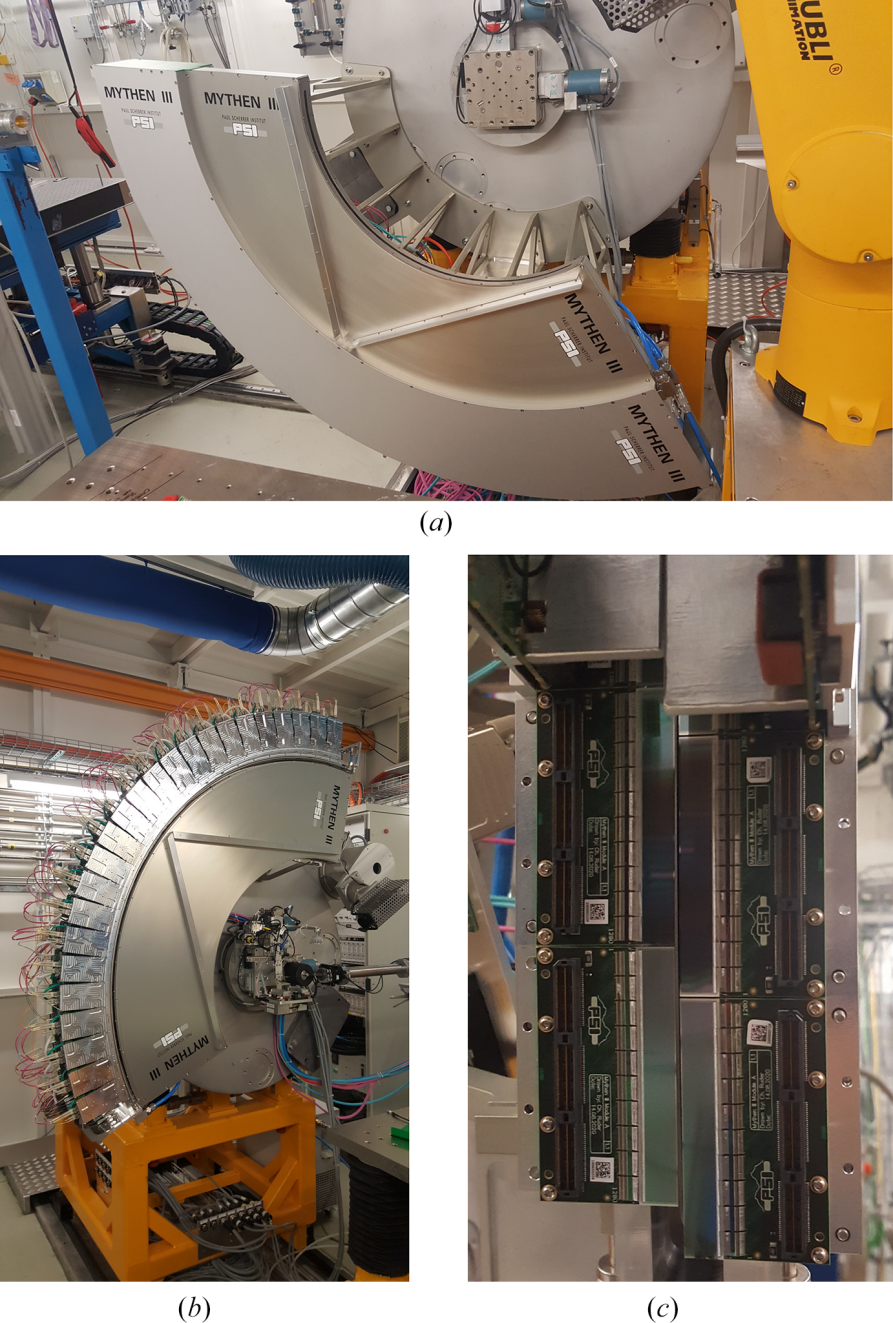
Pictures of the MYTHEN III detector installed on the MS beamline. (*a*) Housing covering 120° in 2θ. (*b*) Modules assembled with all the connections visible. (*c*) Enlargement of the modules tiled close to each other. The spacers mounted on top of the sensor are visible in the top part of the picture. They are needed to avoid collisions between the two rows of the detector. The photons enter from below.

The complete MYTHEN III detector, mounted on the 2θ circle of the diffractometer of the MS beamline at the SLS (Willmott *et al.*, 2013 ▸), is shown in Figs. 3 ▸(*a*) and 3 ▸(*b*). The detector covers a solid angle of 120° and consists of two parallel rows of 24 modules each, shifted by 1° (∼250 strips) between them to eliminate gaps between the modules, as shown in Fig. 3 ▸(*c*). This design allows the detector to cover a wider solid angle of the diffraction cone, thanks to the doubled horizontal acceptance. The absence of gaps in the diffraction pattern means there is no need to move the detector, which saves valuable beam time, especially for time-resolved experiments.

The design of the microstrip sensor has been optimized by reducing the size of the guard rings and minimizing the distance to the dicing lines, allowing the two rows of detectors to be placed as closely together as possible. A spacer, the same size as the sensor, is mounted on the module to prevent collisions between the sensors in the two rows [visible in the top modules in Fig. 3 ▸(*c*)]. The silicon sensors have a thickness of 320 µm in the first row and 450 µm in the second row. The thicker sensor provides higher quantum efficiency above 12 keV, while the thinner sensor offers improved point spread function and reduced parallax.

A total of 48 detector modules (61440 channels) are operated in parallel. Fig. 3 ▸(*b*) shows the detector without its cover, revealing the modules with their power and fiber connections. To ensure stable operation, the detector is water-cooled to maintain room temperature. The distance between the sample and the sensor remains at 76 cm, the same as in MYTHEN II. This configuration yields an angular resolution of 0.0037° based on the 50 µm strip pitch. However, the typical full width at half-maximum (FWHM) for standard profile samples is defined by the capillary and beam characteristics (≥0.018°) rather than by the detector’s resolution (Gozzo *et al.*, 2010 ▸).

At the SLS, MYTHEN III is controlled using an EPICS driver based on *areaDetector* (Wang, 2024 ▸). Alternatively, it can be controlled or integrated into any other control system using the open source C++ and Python application programming interface (API) provided, which is common to all detectors developed at PSI and used at facilities worldwide (Thattil & Fröjdh, 2024 ▸).

## 3. Detector characterization

To determine the average gain, noise and threshold dispersion of MYTHEN III, an energy calibration is carried out. Three different settings, using various feedback voltages and capacitors, have been defined to strike a balance between noise levels and count rate capability, depending on the application’s requirements. These settings are: *Standard*, covering most applications; *High gain* for low energy detection or improved fluorescence discrimination; and *Fast* for high-flux applications. Additionally, the threshold dispersion, count rate capability and temperature stability of a single module were also analyzed.

### 3.1. Energy calibration

To translate the threshold voltages from internal digital to analog converter (DAC) units to the corresponding energies, threshold scans are performed. The detector is exposed to diffused monochromatic X-ray light^1^ on the MS beamline and data are collected as the three global thresholds are scanned. The resulting curves are fitted for each channel with the S-curve model described by Bergamaschi *et al.* (2010 ▸). From this, the inflection point corresponding to the specific X-ray energy, as well as the equivalent noise charge (ENC), are extracted.

The inflection points at various energy levels are used to determine the detector’s linear gain for each setting. The average gain ranges from 0.017 ± 0.001 DAC eV^−1^ to 0.106 ± 0.003 DAC eV^−1^ depending on the settings, as shown in Table 1 ▸. The error represents the gain variation across the more than 60000 channels of the detector, with spreads of 3% for the *High Gain* setting and 5% for the *Standard* and *Fast* settings.

#### 3.1.1. Noise

The electronic noise of the detector is defined by the ENC, *i.e.* the amount of charge needed at the detector input to create an output signal at the end of the analog chain equivalent to the measured noise (Radeka, 1988 ▸). The ENC is typically expressed in electrons and can be converted into energy by noting that, in silicon, the average energy needed to generate an electron–hole pair is 3.6 eV. The noise depends on the settings of the preamplifier and shaper, and it varies with the gain and shaping time of the detector. Specifically, the noise increases as the gain and shaping time decrease. The ENC (r.m.s.) value is determined using the S-curve model described by Bergamaschi *et al.* (2010 ▸). The results are summarized in Table 1 ▸, with the average ENC ranging from 121 ± 8 electrons in the *High gain* setting to 372 ± 22 electrons in the *Fast* setting. The errors represent the standard deviation of the noise distribution of all the channels of the detector.

For optimal performance in single photon counting detectors, the threshold is ideally set at half the photon energy. To avoid noise counts, the minimum threshold should be approximately 5 × ENC, which corresponds to a minimum detectable energy of ∼10 × ENC. Consequently, the minimum detectable energies are 5.0 ± 0.3 keV for *Standard*, 4.3 ± 0.3 keV for *High Gain* and 13.4 ± 0.8 keV for *Fast* settings. Lower energies can still be detected by reducing the threshold down to ∼3 × ENC, though this comes at the cost of some noise counts. Alternatively, a threshold higher than half the photon energy can be used, though this will result in a loss of quantum efficiency. For example, the *Fast* setting can be used to detect photon energies ≥8 keV with a threshold ≥4 keV.

The ENC also plays a crucial role in distinguishing between fluorescence and elastically scattered photons. As a rule of thumb, the threshold should be set 3 × ENC away from both the fluorescence emission and the primary beam energy. This means that the minimum separation between the fluorescence emission and the scattered beam energy is about ∼6 × ENC, which corresponds to 3.0 ± 0.2 keV for *Standard*, 2.6 ± 0.2 keV for *High Gain* and 8.0 ± 0.4 keV for *Fast* settings. In the *Standard* setting, the improvement over MYTHEN II is almost a factor of two.

#### 3.1.2. Threshold equalization

The threshold dispersion of the detector reflects its response uniformity across all channels and impacts the quality of the flat-field correction, which is essential for accurate experimental data. To improve this uniformity, the detector undergoes a process called trimming. In this process, all thresholds are equalized using the six individual trimbits per comparator, ensuring that all comparators trigger at the same input signal level. This threshold equalization enhances data quality, especially when dealing with fluorescence light emitted by the sample. A flatter detector response simplifies flat-field corrections, even when there is a spectral difference between the reference beam and the actual measurement.

The trimming method used is the same as that described by Kraft *et al.* (2009 ▸). First, a threshold scan is performed under monochromatic radiation to determine the trimming threshold, which will ultimately correspond to the trimming energy. Next, a scan of the trimbit current is conducted to define the size of the trimbit LSB (least significant bit). Finally, the trimbits are scanned to determine the optimal value for each channel. Trimming primarily corrects for gain and offset mismatches between channels and is typically performed on only one comparator, as the analog chain is shared. Any remaining differences can be corrected by calibrating the global threshold of the chip individually for each comparator.

The trimming is performed at several energy levels (on the MS beamline, seven energies ranging from 7.2 keV to 22 keV). The calibration for other threshold values is achieved by interpolating or extrapolating the global threshold, trimbit current and trimbit values.

Trimming typically reduces the threshold dispersion by a factor of ∼10, as shown in Table 1 ▸. For the *High gain* setting, the average threshold dispersion of the entire detector with 24 modules is reduced from 430 ± 30 eV in the untrimmed case to 33 ± 10 eV after trimming. The error bar represents the variation between the 48 modules of the detector.

### 3.2. Temperature stability

The system’s temperature affects both the sensor and the readout electronics by influencing the charge carrier mobility and the internal resistance of the on-chip transistors. These factors, in turn, impact the effective feedback reference voltages, which define the detector’s gain and noise levels. To ensure stable operation and predict detector performance under various experimental conditions, the temperature stability of the calibration was studied by repeating the energy calibration of a module at different gain settings, with temperatures ranging from 10°C to 40°C. As the temperature increases, the gain of a module decreases by 0.40 ± 0.19% °C^−1^ in the *Standard* setting and by 0.31 ± 0.32% °C^−1^ in the *Fast* setting. Similarly, the ENC increases with temperature, rising by 0.44 ± 0.15% °C^−1^ in the *Standard* setting. The errors represent the variation between the channels.

The results suggest that sensor effects, such as changes in conductivity or charge carrier mobility, play a minor role compared to the impact of leakage current and changes within the ASIC. However, the influence of temperature on detector performance becomes significant when temperature variations exceed 10°C, and re-calibration of the detector is recommended for such large temperature changes.

### 3.3. Count rate capability

With the expected increase in photon flux at the SLS 2.0 (Streun *et al.*, 2018 ▸), single photon counting detectors will face new challenges due to the higher count rate requirements of the beamlines. Although the increase in photon flux on the MS beamline is not as significant as for beamlines that rely on beam coherence, the count rate capability remains a critical performance metric. With the high level of accuracy desired for many X-ray powder diffraction experiments (<1%), any count rate corrections present a certain level of inaccuracy and should be avoided. With MYTHEN II, a deviation from linearity was already visible at 100 kphotons s^−1^ strip^−1^, which can be achieved with several samples. Hence the effort in the improvement of the count rate capability.

The primary limitation of single photon counting detectors at high photon flux is caused by signal pile-up, where overlapping analog signals reduce counting efficiency. This effect can be modeled by defining a dead time τ_d_, which depends on the shaping of the analog chain. The dead time represents the minimum interval required between two photon hits for them to be recorded separately (Leo, 1994 ▸; Knoll, 2010 ▸). If two photons arrive too close together in time, the second hit will be lost.

The paralyzable counter model is useful for describing the behavior of a photon counter like MYTHEN III, as its preamplifier and shaper are continuously sensitive to incoming charge. The probability *p* of signal pile-up involving *n* photons can be modeled as a function of the photon flux ϕ_0_,
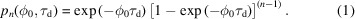
For a standard single photon counting detector with a single threshold, the observed flux is given by ϕ_obs_ = *p*_1_ ϕ_0_. In the case of MYTHEN III, the three thresholds can be adjusted to detect the pile-up of up to three photons arriving within the dead time interval τ_d_.

The lowest threshold is set to half the photon energy *E*_γ_, allowing the detection of the first photon. The second and third thresholds are set between one (two) and two (three) times *E*_γ_, respectively, so that the second (third) comparator only triggers when a second (third) photon arrives simultaneously, causing pile-up. After determining the dead time of the detector, the observed flux can be calculated as
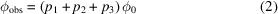
and ε_tot_ = *p*_1_ + *p*_2_ + *p*_3_ can be defined as the count rate efficiency (Fröjdh *et al.*, 2024 ▸).

**Figure 4:**
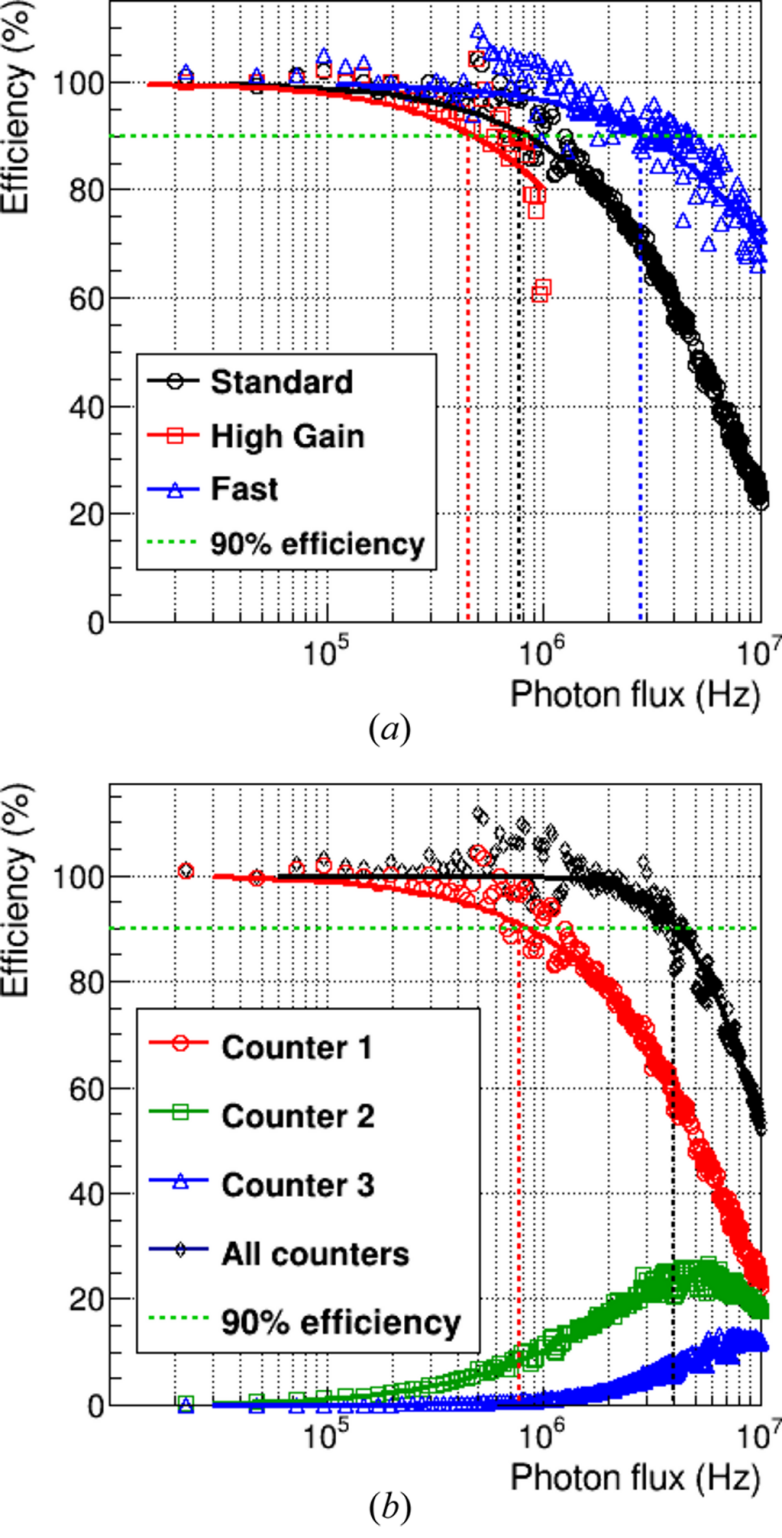
Detection efficiency at 12 keV (*a*) for the different settings and (*b*) for the three counters and their sum with the *Standard* setting (thresholds at 0.5 × *E*_γ_, 1.025 × *E*_γ_ and 1.82 × *E*_γ_, respectively). The dashed lines represent the photon flux at which the count rate efficiency equals 90%.

To measure the count rate efficiency, the detector is placed in a direct monochromatic X-ray beam. The 4 mm wide 12 keV beam on the MS beamline illuminates around 80 strips of the module. The beam intensity is varied using silicon filters of different thicknesses, while the reference photon flux ϕ_0_ is monitored by the beamline instrumentation. The flux ϕ_obs_ recorded by the MYTHEN III module at each attenuation step is then compared to ϕ_0_ to estimate the loss in detection efficiency as a function of beam intensity. By fitting the data with the paralyzable counter model [see equation (2)], the dead time τ_d_ can be extracted. The measurement was repeated for all the settings. Fig. 4 ▸(*a*) shows the detection efficiency for one counter for the three different settings. Fig. 4 ▸(*b*) presents the same plot for the three counters and their combined total, using the *Standard* setting. The threshold for the first counter was set to half the photon energy, as in ideal conditions. The thresholds for the second and third comparators were adjusted so that their rate efficiency curves fit the same τ parameter as the first counter (1.025 × *E*_γ_ and 1.82 × *E*_γ_, respectively). This ensures that the sum of the three counters does not overestimate the number of impinging photons, allowing for maximum efficiency while keeping it below 100% (within the margin of error).

The curves have been fitted using the paralyzable counter model, which accounts for the expected number of pile-up photons at each threshold. Table 2 ▸ lists the photon flux levels at which the count rate efficiency drops below 90%. The results for *High gain* setting are not included, as the analog chain saturates in the case of pile-up.

The maximum count rate at 90% efficiency, using the *Fast* setting and all three counters for pile-up tracking, exceeds 10 Mphotons s^−1^ strip^−1^. However, the count rate capability of the MYTHEN III module is not as fast as the one reported by Andrä *et al.* (2019*a* ▸) for the MYTHEN 3.0.2 prototype. This is due to the slower *Fast* setting defined for the MYTHEN III module, which was adjusted to limit bandwidth and reduce common-mode noise in the ten-chip assembly – a challenge more pronounced than for smaller prototypes. Despite this, MYTHEN III can handle much higher photon flux levels than most commercially available photon counting detectors, outperforming MYTHEN II by an order of magnitude.

### 3.4. Frame rate

The MYTHEN III readout electronics are significantly faster than those used for MYTHEN II thanks to several key improvements: a faster readout clock (100 MHz compared with 10 MHz), increased parallelization (four serial outputs instead of one per chip) and higher data throughput (10 Gbps UDP stream versus 100 Mbps TCP/IP data transfer). Since the data streaming outperforms the readout speed, the acquisition can run continuously at the maximum speed, with no need for a burst mode, as long as the network infrastructure and the data acquisition (DAQ) system PC can support the data throughput.

The maximum frame rate of the detector scales both with the number of counters being read out and with the bit depth, which can be configured to 24 (streamed out as 32 bit), 16 or 8 bits (see Table 3 ▸).

Additionally, the data streaming remains fully parallel even in multi-module systems, regardless of detector size. On the MS beamline of the SLS, the modules are connected to the data storage via a switch with a maximum bandwidth of 40 Gbps. Therefore, when reading out the whole 48-module detector, the maximum frame rate must be reduced by more than an order of magnitude compared to the ideal values listed in Table 3 ▸. Higher frame rates can be achieved by reducing the number of modules to be read out (region of interest) or by upgrading the DAQ system.

### 3.5. Gateability

In pump–probe experiments [see *e.g.* Laulhé *et al.* (2012 ▸)], single photon counting detectors can be operated in stroboscopic mode by toggling the acquisition window without reading the detector between successive gates. This makes it possible to achieve a time resolution better than the maximum frame rate. For MYTHEN III, the achievable time resolution is primarily determined by the shaping of the analog signal. The three counters can be gated independently, creating different time slots for pump–probe experiments. This allows for more efficient measurements, such as reducing the total measurement time by a factor of three by using different delays or acquiring pumped and unpumped data in parallel (Burian *et al.*, 2020 ▸).

**Figure 5:**
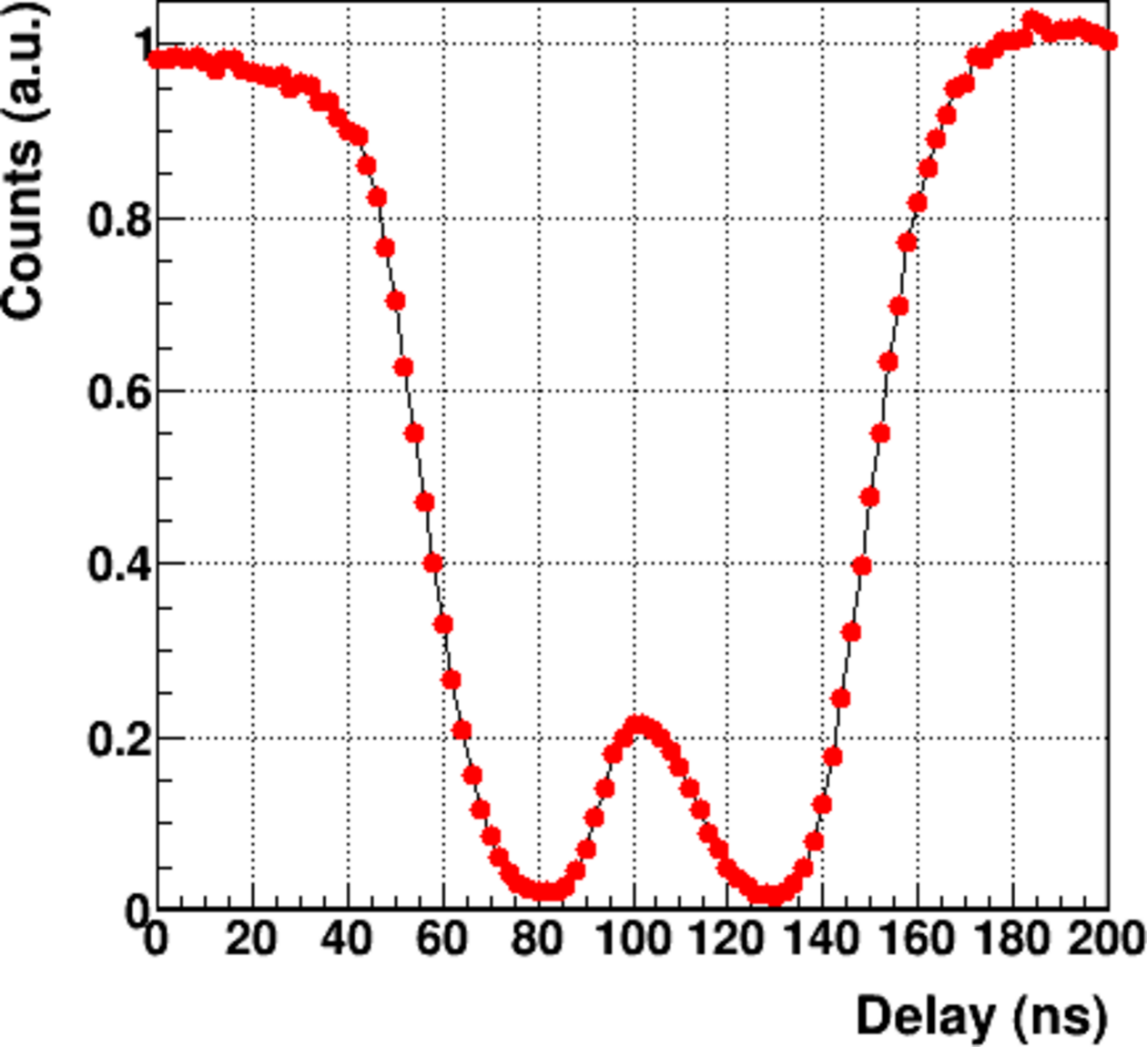
Delay scan using a 10 ns wide gate and the *Fast* setting for 8 keV photons with the threshold set at 4 keV.

The hybrid-mode filling pattern of the SLS is characterized by a bunch train of 390 filled buckets spaced by 2 ns plus a single bunch of higher intensity (*Camshaft*) placed in the center of the 100 ns gap. The machine provides the so-called *Camshaft* signal, which is synchronized with the revolution period of the electrons in the SLS storage ring (Schlott *et al.*, 2004 ▸). Fig. 5 ▸ shows the total number of counts of a MYTHEN III module gated for 10 ns as function of the delay to the delay from the *Camshaft* signal. The data were collected on the superXAS beamline of the SLS at 8 keV using the *Fast* setting, with the threshold set at 4 keV (Smolentsev *et al.*, 2014 ▸). The isolated *Camshaft* bucket located in the middle of the 180 ns gap used in this filling pattern is well resolved, with a FWHM of ∼35 ns. When the gate width is subtracted quadratically, the result is an effective time resolution of ∼33 ns. This is further confirmed by the ratio between the intensity of the 500 MHz filling pattern (where single bunches separated by 2 ns cannot be resolved) and the isolated *Camshaft* bunch, which should be four times more intense than the others.

## 4. X-ray powder diffraction

Data acquisition of powder diffraction patterns using the novel MYTHEN III detector was performed on the MS beamline of the SLS following a process similar to previous measurements with the MYTHEN II. Typical acquisitions involved two or more detector positions (to cover the gaps between the modules and potential bad channels), with samples loaded in a glass capillary rotating on its *X* axis at a frequency of 1–4 Hz. The beam had dimensions of 4.0 mm × 1.0 mm and energies in the range 5.6–32 keV. Flat-field and angular calibrations were derived and applied to primary data as described by Bergamaschi *et al.* (2010 ▸). Since the geometric features of MYTHEN II and MYTHEN III, including sensor-strip dimensions and distance to the sample, remained unchanged, the geometrically related characteristics in the powder diffractogram, such as intrinsic FWHM and pixel resolution, also remained consistent. Comparisons of profile standards collected under similar conditions did not reveal discrepancies in profile shapes.

**Figure 6:**
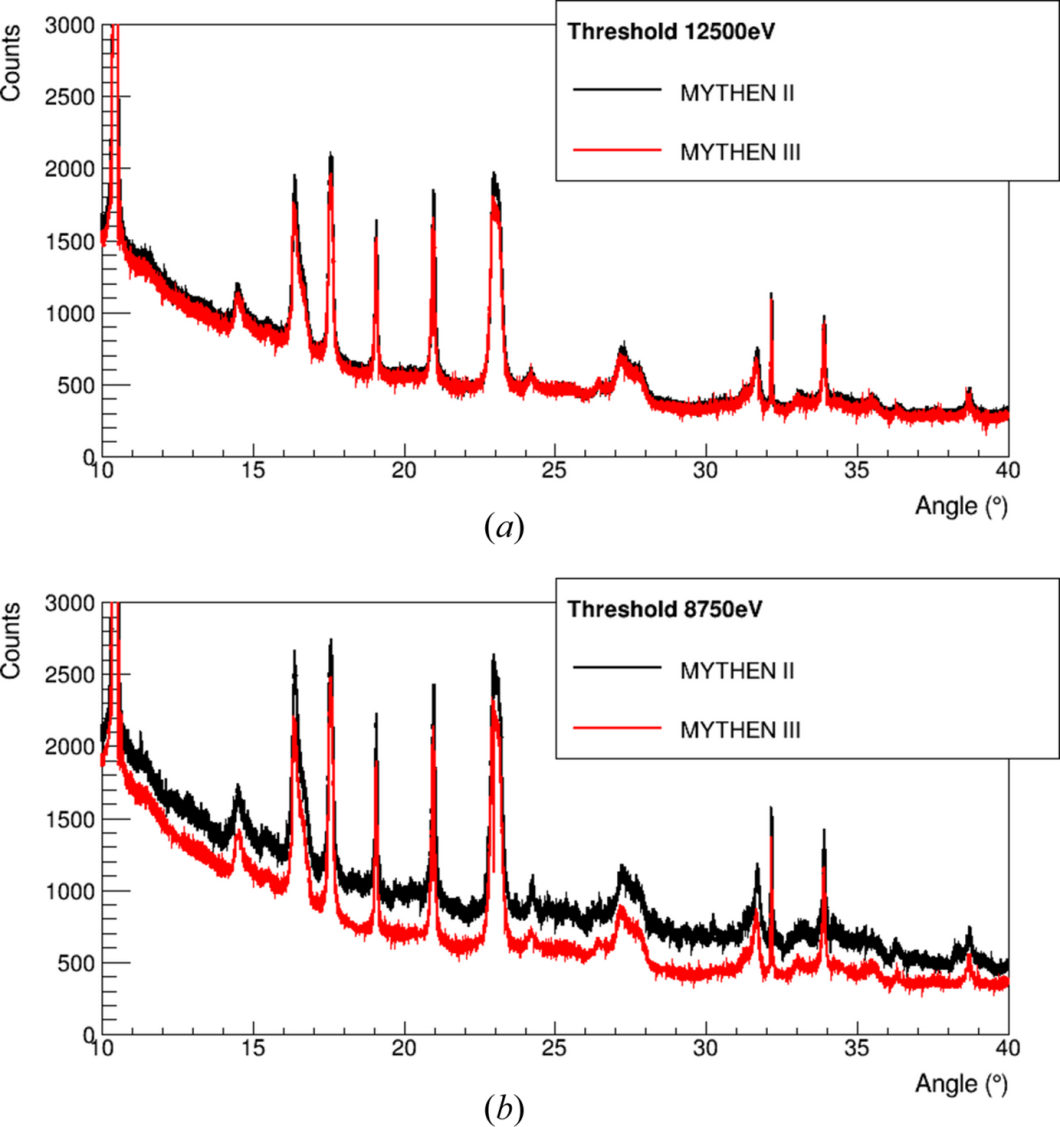
Diffractograms of a copper oxalate powder collected at 17.5 keV with MYTHEN II and MYTHEN III. (*a*) Comparator threshold set at 12500 eV, in order to maximize the copper fluorescence background suppression. (*b*) Comparator threshold set at 8750 eV as in ideal conditions. The lower noise and improved threshold dispersion of MYTHEN III effectively suppress the copper fluorescence background, with less than 1 keV difference between the threshold and the energy of the fluorescence radiation.

Fig. 6 ▸ shows the diffractograms of a copper oxalate powder contained in a 0.3 mm capillary spun at 1 Hz. The data were acquired at 17.5 keV using different thresholds simultaneously under the same conditions for both MYTHEN II and MYTHEN III. In Fig. 6 ▸(*a*), the comparator threshold was set to 12500 eV, meeting the requirement that the threshold be at least 3 × ENC above the 8.05 keV copper *K*α fluorescence line for both MYTHEN II and MYTHEN III (see Section 3.1.1). As a result, the data are highly comparable. Fig. 6 ▸(*b*) illustrates that, due to lower noise and improved threshold dispersion, the MYTHEN III pattern is significantly less affected by fluorescence background, even when the threshold is set at 8750 eV. In this case, the MYTHEN III comparator threshold is less than 2 × ENC above the fluorescence line and lower than the copper *K*β lines at 8.9 keV. Despite this, in contrast to MYTHEN II, the MYTHEN III diffractogram closely resembles the one obtained with a higher threshold, showing minimal contamination from the fluorescence background. These improved performance characteristics facilitate selecting the appropriate X-ray energy for samples containing multiple elements, where identifying a suitable beam energy and threshold combination can sometimes be challenging.

One of the main goals of the MYTHEN III upgrade was the ability to collect data across elemental edges, allowing for the characterization of short-range element-specific structural features and long-range average order within the crystalline structure, taking advantage of the sharp changes in the scattering factors in the proximity of such edges (Stragier *et al.*, 1992 ▸). Such a method could also be employed in specific cases (with low dilution) with MYTHEN II but required a much more complex data acquisition protocol (Knorpp *et al.*, 2021 ▸). In fact, diffraction is often hindered by the large fluorescent signal emitted when the beam energy exceeds the absorption edge of specific elements in the sample. The detector simultaneously collects both elastically scattered photons and a significant angle-independent fluorescent signal at lower emission energy, typically 10–15% lower than the incident energy for *K*α lines. Under common measurement conditions, this fluorescent signal cannot be sufficiently suppressed by simply adjusting the threshold between the energies, with MYTHEN II and even with MYTHEN III with improved ENC.

To compare the performance of MYTHEN II and MYTHEN III for on-edge measurements, the same experiment was conducted just above the molybdenum *K*-edge, using both detectors with a sample containing molybdenum emitting *K*α fluorescence at 17.480 keV. The experimental conditions were kept identical, with a beam energy of 20.05 keV and a detector threshold set at 15 keV, although the tests were conducted at different times – before and after the detector upgrade. A freshly prepared sample of molybdenum acetate was ground and filled into a 3 mm glass capillary for the test. In both cases, a flat field with comparable statistics was acquired under the same experimental conditions.

**Figure 7:**
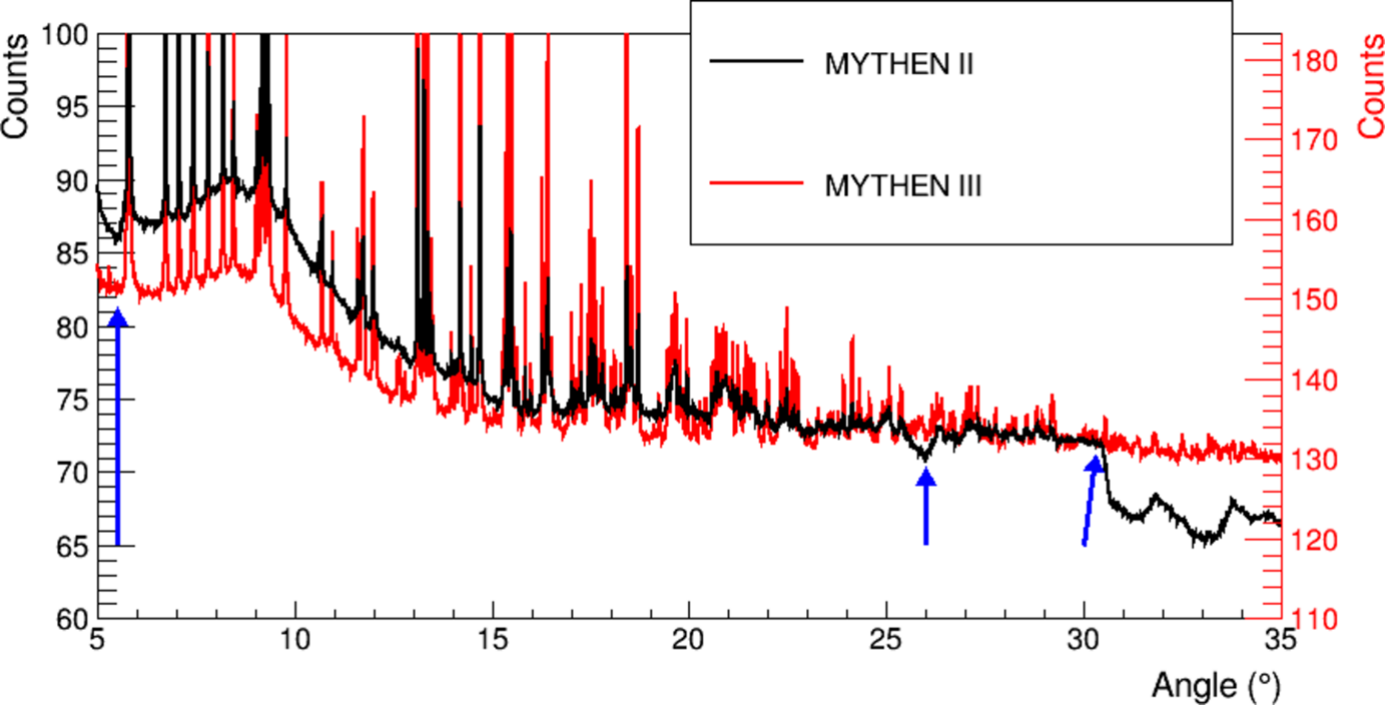
Diffractograms for molybdenum acetate, collected above the Mo *K*-edge with MYTHEN II and MYTHEN III. The data have been normalized to the total number of counts to improve comparability. The data were collected using 42 different detector positions, with each position measured for 15 s. The diffractogram collected with the older detector (shown in black) exhibits discontinuous background features – for example, dips just before sharp peaks at 5.5°, 26° and slightly above 30° – as indicated by the blue arrows. These artifacts are due to higher threshold dispersion, which could not be corrected by flat-field calibration. Since the measurements for MYTHEN II and MYTHEN III took place at different times (before and after the detector upgrade, respectively), the differences in background may be influenced by variable factors in the setup. These factors include varying contributions of air scattering and capillary scattering relative to the sample’s maximum intensity during the two experiments.

A portion of the two diffractograms, after angular conversion and flat-field correction, is reported in Fig. 7 ▸. The results highlight the improved background characteristics with MYTHEN III (red), while the main diffraction peaks appear similar for both detectors. Due to variations in the responses of the different channels and modules to the two different energies, the diffractogram collected on-edge with the MYTHEN II detector contains a biased background that cannot be improved by longer acquisitions, which only reduce the statistical noise component. The flat-field calibration, which inherently differs from the experimental conditions due to the absence of fluorescence from the sample, also fails to correct these artifacts adequately. On the other hand, the reduced threshold dispersion of MYTHEN III results in more consistent channel and module responses, leading to better overall data quality.

## 5. Beam monitoring

Given the extremely high accuracy demonstrated by powder diffraction data acquired with MYTHEN III [see *e.g.* Spiliopoulou *et al.* (2021 ▸), Bertolotti *et al.* (2022 ▸) and Bertolotti *et al.* (2024 ▸)], it has become increasingly important to correct for beam instabilities that could affect data quality. Additionally, knowing the statistical error of the beam intensity monitor is crucial, so that it can be appropriately factored into the data error – although this is usually negligible except for weakly scattering samples.

Beam stability can be monitored by detecting the elastic scattering of the primary beam by air, which is proportional to the beam’s intensity. Compared to traditional beam monitors in transmission, such as ionization chambers, using a single photon counting detector like MYTHEN to detect the scattered radiation offers several advantages. This method avoids beam absorption and allows for monitoring the beam over a wide range of intensities, while also synchronizing the monitor with the diffraction detector with minimal delay 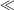
10^−7^ s, only limited by the cable length.

Air scattering is sufficiently strong at all energies to generate a total monitor signal (sum of 1280 channels) well above one million counts per second, resulting in a relative error 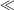
10^−3^. For an attenuated beam, a longer exposure time can reduce the error, as it decreases proportional to the square root of the exposure time (and the number of detected photons).

### 5.1. Beam intensity monitor

**Figure 8:**
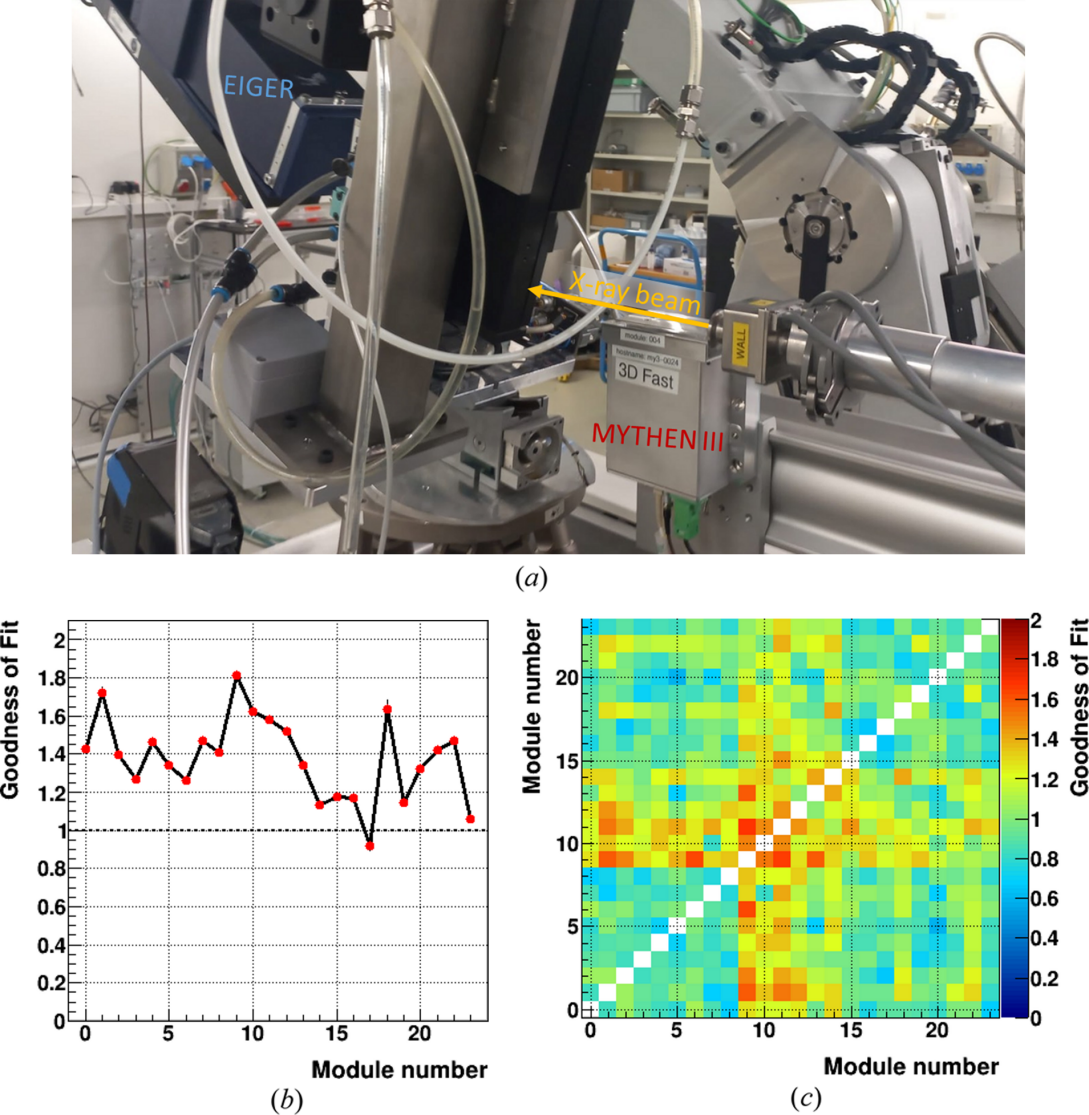
(*a*) Picture of a MYTHEN III module used as beam intensity monitor in combination with EIGER for multi-kilohertz *operando* experiments. (*b*) GoF for each module on the average of 16 repeated 30 s acquisitions when scaled with the MYTHEN II module monitor. (*c*) GoF for each module on the average of 16 repeated 30 s acquisitions when scaled by all other detector modules. When scaling one module with itself, the GoF yields 0, resulting in the diagonal cut in the graph.

On the MS beamline, an additional MYTHEN module is routinely placed 20–30 mm below a short segment of the direct beam traveling through air, with the sensor positioned parallel to the beam, to monitor beam intensity. Alternatively, a low-absorption sample, such as a Kapton foil, can be used to enhance scattering or for experiments in vacuum. Currently, a MYTHEN II module is used for the measurements presented in this paper, but it will be replaced with MYTHEN III for SLS 2.0, as both detectors share identical geometry and are expected to perform equivalently. Due to its superior frame rate, MYTHEN III has already been used for beam monitoring in time-resolved experiments [see *e.g.* Hocine *et al.* (2020 ▸)] in the configuration shown in Fig. 8 ▸(*a*), despite not yet being fully integrated in the beamline controls.

The diffraction detector modules are synchronized using a trigger cable, resulting in negligible delays, while the MYTHEN II module used for beam monitoring is controlled by software, which introduces jitters of the order of milliseconds. However, these jitters are irrelevant when the primary beam varies at frequencies of a few hertz.

The effectiveness and precision of beam monitoring were tested by taking repeated long acquisitions of radiation scattered by an amorphous sample and normalizing the sum of the counts from each module against the beam monitor’s total count. The test uses the weighted average of the total counts for each module after normalization and evaluates the goodness of fit (GoF) of the weighted average. The GoF is the ratio of the effective dispersion of the averaged values to the implied dispersion of the weighted average. More details on this method are provided in Appendix *A*[App appa].

The GoF is expected to be 1 under ideal conditions, which, for a counting detector, would occur if Poisson statistics were the only source of variation. However, at extremely high counting statistics for both the data and the monitor, errors from Poisson statistics are of the order of 10^−5^. Consequently, even minimal instabilities of a similar magnitude, whether originating from the detector or the beam position, result in a measured GoF of 1.2–1.5. Fig. 8 ▸(*b*) shows the GoF calculated for the first 24 modules of the detector installed at the SLS. The GoF approaches the ideal value when the modules are normalized against each other [Fig. 8 ▸(*c*)], thanks to the better synchronization.

### 5.2. Polarization monitor

The anisotropic angular intensity distribution around the beam direction originating from the polarization of synchrotron X-rays can be exploited to measure the average polarization of the beam. In experiments utilizing differential magnetic contrast (X-ray magnetic dichroism), a birefringent phase retarder is inserted into the beam after the monochromator. In the X-ray regime, birefringence occurs in crystals like silicon or diamond, which meet the conditions for dynamical diffraction, especially near the absorption edge of the material. By positioning the phase plate diffraction plane at 45° relative to the horizontal plane of the synchrotron, the two waves propagating through the crystal can be dephased, creating circular polarization and enabling the rotation of incident polarization from horizontal to vertical (Giles *et al.*, 1994 ▸; Scagnoli *et al.*, 2009 ▸).

**Figure 9:**
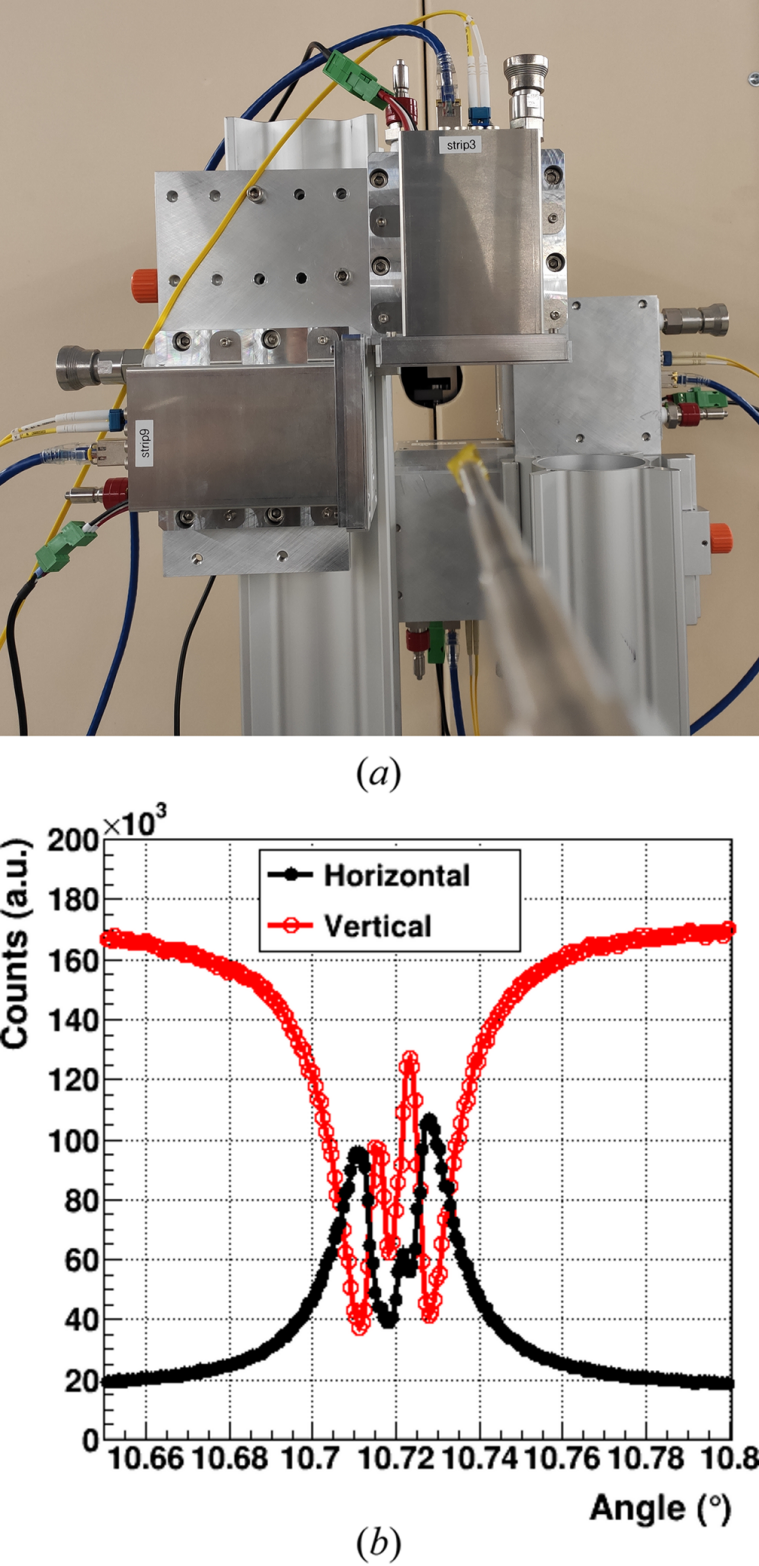
(*a*) Picture of the setup using two pairs of MYTHEN III modules placed on top and on the side of the beam to detect the scattered radiation and measure the beam polarization. (*b*) Integrated counts of two modules as a function of the angle of the diamond phase plate acquired at 7.2 keV.

Two MYTHEN III modules, mounted to detect air scattering in the horizontal and vertical directions, respectively [as shown in Fig. 9 ▸(*a*)], allow for real-time monitoring of the beam’s polarization state by comparing their relative signals.

Fig. 9 ▸(*b*) illustrates the normalized intensity of the two detectors as a function of the misalignment of the diamond phase plate with respect to the Bragg angle, taken at the iron *K*-edge 7.112 keV on the cSAXS beamline at the SLS. At the start of the measurement, the beam is horizontally polarized, resulting in mostly vertical scattering (red curve) and minimal horizontal scattering (black curve). As the rocking angle approaches the Bragg peak, the polarization begins to rotate, reaching vertical polarization around 10.71°, where the red curve reaches a minimum and the black curve a maximum. At the Bragg position, around 10.72°, circular polarization is achieved. Beyond this point, the polarization transitions back to linear (first vertical, then horizontal) as the crystal is rocked further.

Unfortunately, a combination of the two channels does not straightforwardly represent the polarization of the X-ray beam. For a quantitative determination, a polarimeter is necessary. However, comparing the MYTHEN III curves with simulations of the polarization state following Giles *et al.* (1994 ▸) easily allows for identifying the positions of the diamond phase plate that ensure a circular polarization exceeding 95%, which is more than sufficient for experiments utilizing magnetic contrast.

This setup is particularly compact and easy to use compared with other existing more accurate systems, *e.g.* Manzanillas *et al.* (2024 ▸). It provides immediate feedback on the polarization state and has been successfully employed in several differential magnetic contrast experiments at the SLS (Apseros *et al.*, 2024 ▸).

## 6. Conclusion

The newly installed MYTHEN III detector on the MS beamline at the SLS meets all the specifications required for the upcoming SLS 2.0 upgrade. Its low noise, minimal threshold dispersion, high count rate capability and dead-time-free readout with fast frame rates ensure that it is well prepared to handle the demands of future experiments.

Compared with MYTHEN II, MYTHEN III offers significant improvements in all key performance metrics. The minimum noise level has been reduced from 700 eV to 450 eV, enabling the detection of lower X-ray energies and better fluorescence suppression. The trimmed threshold dispersion has been reduced by a factor of four, making the detector response much more uniform and enhancing flat-field quality.

In terms of count rate capability, MYTHEN III has made a substantial leap, reaching 0.5–2.9 MHz using one counter and 4–11 MHz with pile-up tracking using three counters (compared with 0.1–1 MHz with MYTHEN II). This allows for powder diffraction experiments with significantly higher photon flux. Additionally, the continous frame rate of MYTHEN III is approximately 400 times faster, enabling time-resolved measurements without dead time and offering a time resolution of 33 ns – critical for studying various physical processes.

While MYTHEN III was developed specifically to meet the requirements of the MS beamline at the SLS, it can also be deployed at other facilities looking to upgrade their existing MYTHEN II systems, *e.g.* Haverkamp & Wallwork (2009 ▸), Thompson *et al.* (2011 ▸), Fauth *et al.* (2015 ▸), Carvalho *et al.* (2016 ▸), Du *et al.* (2016 ▸) and Osaka *et al.* (2019 ▸), or to equip their powder diffraction stations with a position-sensitive detector. Similarly, the improved performance can also bring advances for experiments using laboratory diffractometers, where strip detectors are largely used (Faske & Donner, 2018 ▸; Thomae *et al.*, 2019 ▸; Hodzic *et al.*, 2022 ▸). Additionally, MYTHEN III extends its applicability to other areas, such as beam and polarization monitoring. It is also expected to replace MYTHEN II in many energy-dispersive spectrometers where microstrip detectors are currently in use (Kleymenov *et al.*, 2011 ▸; Szlachetko *et al.*, 2012 ▸).

In addition, MYTHEN III has served as a testing ground for several solutions that will be implemented in MATTERHORN (Fröjdh *et al.*, 2024 ▸), the next-generation single photon counting pixel detector under development at PSI. Specifically, MATTERHORN features four independent comparators and counters, and it relies on fast shaping and pile-up tracking to support the high photon fluxes expected with the upgraded SLS 2.0.

We are confident that the advanced capabilities of MYTHEN III will be exploited in numerous scientific experiments, as was the case with its predecessor, which has been a cornerstone of experimental research for over 15 years.

## Figures and Tables

**Table 1:** Calibration results for the MYTHEN III detector in three different gain settings at room temperature The measurements were taken at 9 keV for the *Standard* and *High gain* settings and at 12.6 keV for the *Fast* setting, with a 320 µm thick Si sensor. The errors in Gain and ENC reflect the variation across the 60000 channels, while the error in the threshold dispersion represents the spread between the 48 modules

			Threshold dispersion (eV)	
Setting	Gain (DAC eV^−1^)	Noise ENC (electrons)	Untrimmed	Trimmed
*Standard*	0.040 ± 0.002	138 ± 9	590 ± 40	55 ± 18
*High gain*	0.106 ± 0.003	121 ± 8	430 ± 30	33 ± 10
*Fast*	0.017 ± 0.001	372 ± 22	840 ± 140	40 ± 14

**Table 2:** List of the achievable count rates at 90% count rate efficiency for one or three counters when using the pile-up model in equation (1) and equation (2) and the respective dead times τ_d_ The errors in τ_d_ represent the uncertainty in the fit and are propagated to the achievable count rates.

		Rate (kphotons s^−1^ strip^−1^)	
Setting	τ_d_	One counter	Three counters
*Standard*	126 ± 25 ns	750 ± 10	4000 ± 500
*High gain*	220 ± 80 ns	450 ± 50	–†
*Fast*	36 ± 10 ns	2800 ± 600	11000 ± 2000

† Data with three counters are missing for the *High gain* setting, because the analog chain saturates in cases of pile-up.

**Table 3:** Maximum frame rate of MYTHEN III as a function of the number of counters and of the bit depth These numbers neglect possible limitations arising from the DAQ computing and network system in cases of large detectors.

	24 bit	16 bit	8 bit
Three counters	40 kHz	60 kHz	120 kHz
Two counters	60 kHz	90 kHz	180 kHz
One counter	120 kHz	180 kHz	360 kHz

## Footnotes

1 Perfect homogeneous illumination can only be achieved by moving the detector in very small increments and restricting acquisition to one small arc, so that every element of the detector is actually exposed to the same total illumination. Due to the 120° range and using a 25–30° arc, in the MS installation this means moving the detector by 160°, in steps of 0.1° or smaller. However, for the purpose of energy calibration, it is sufficient that all the strips of a module are illuminated with approximately the same intensity.

## Appendix A Goodness-of-fit

To understand the statistical method used to evaluate the performance of the beam intensity normalization, consider having *N* independent measurements *y*_*j*_ of the same quantity *a*, each with a different estimated standard deviation *e*_*j*_. The best estimate (in the maximum likelihood sense) of *a* is obtained by minimizing

The minimization yields the best value,

This is exactly the variance-weighted arithmetic mean (weighted average). The (co)variance of *a*_*_ is estimated (Giacovazzo, 2011 ▸; Press *et al.*, 1992 ▸) as

where the second factor is the squared goodness of fit (GoF) and the first factor is the ideal variance estimate of the weighted average, while the second term takes into account the possible excess dispersion of the data points due to underestimating *e*_*j*_ when not all sources of error are properly taken into account.

The measurements *y*_*j*_ are (sums of) counts collected in a fixed time, and as such they are described by Poisson statistics, *i.e.* a reading *y*_*i*_ is assumed to indicate a Poisson distribution with average *y*_*i*_ and variance *y*_*i*_. However, Mighell (1999 ▸) showed that this is statistically indistinguishable from a normal distribution with average *y*_*i*_ + min[*y*_*i*_, 1] and variance *y*_*i*_ + 1. Now, when processing the data, even by a simple operation like dividing by the monitor (that in this case is a fixed-time count as well), the variance of the result can be easily and rigorously estimated if all involved variables are normally distributed, and the result will still be normally distributed. In this way, the usage of the χ^2^ distribution and the formalism of the GoF above are fully justified.
